# The Arabidopsis *ORGAN SIZE RELATED 2* is involved in regulation of cell expansion during organ growth

**DOI:** 10.1186/s12870-014-0349-5

**Published:** 2014-12-10

**Authors:** Zhixiang Qin, Xiao Zhang, Xiaoran Zhang, Guanping Feng, Yuxin Hu

**Affiliations:** Key Laboratory of Plant Molecular Physiology, Institute of Botany, Chinese Academy of Sciences, Beijing, 100093 China; University of Chinese Academy of Sciences, Beijing, China; Key Laboratory for Biodiversity Science and Ecological Engineering, School of Life Sciences, Jinggangshan University, Ji’an, Jiangxi 343009 China; National Center for Plant Gene Research, Beijing, China

**Keywords:** Arabidopsis, Cell expansion, Cell proliferation, OSR, Organ size

## Abstract

**Background:**

In plants, the growth of an aerial organ to its characteristic size relies on the coordination of cell proliferation and expansion. These two different processes occur successively during organ development, with a period of overlap. However, the mechanism underlying the cooperative and coordinative regulation of cell proliferation and expansion during organ growth remains poorly understood.

**Results:**

This study characterized a new Arabidopsis *ORGAN SIZE RELATED* (*OSR*) gene, *OSR2*, which participates in the regulation of cell expansion process during organ growth. *OSR2* was expressed primarily in tissues or organs undergoing growth by cell expansion, and the ectopic expression of *OSR2* resulted in enlarged organs, primarily through enhancement of cell expansion. We further show that *OSR2* functions redundantly with *ARGOS-LIKE* (*ARL*), another *OSR* gene that regulates cell expansion in organ growth. Moreover, morphological and cytological analysis of triple and quadruple *osr* mutants verified that the four OSR members differentially but cooperatively participate in the regulation of cell proliferation and cell expansion and thus the final organ size.

**Conclusions:**

Our results reveal that *OSR2* is functional in the regulation of cell expansion during organ growth, which further implicates the involvement of OSR members in the regulation of both cell proliferation and expansion and thus the final organ size. These findings, together with our previous studies, strongly suggest that OSR-mediated organ growth may represent an evolutionary mechanism for the cooperative regulation of cell proliferation and expansion during plant organogenesis.

**Electronic supplementary material:**

The online version of this article (doi:10.1186/s12870-014-0349-5) contains supplementary material, which is available to authorized users.

## Background

For multicellular organisms, organ size is a fundamental attribute of body morphology [1]. In animals, the final size of an organ is primarily determined by cell growth, proliferation, and apoptosis. These processes are primarily mediated by two major pathways: the target of the rapamycin (TOR) pathway that regulates cell growth and the Hippo pathway that coordinately controls cell growth, proliferation, and apoptosis [2,3]. In plants, because cell apoptosis does not generally contribute to morphogenesis in most organs [4], the development of an organ to its characteristic size depends mainly on cell proliferation and expansion [5,6]. Recent studies in Arabidopsis have identified a number of genes involved in the regulation of either cell proliferation or cell expansion that are now known to affect final organ size [7,8]. However, many such factors appear to be involved in multiple pathways to affect cell number or size [8], suggesting that the mechanisms of organ-size control in plants are more complicated than those in animals [1,9]. This might be partly because plants are immobile and have to be highly responsive to the ever-changing environments [1].

During the development of plant determinate organs such as leaves and floral organs, cell proliferation and expansion occur at two distinct but overlapping phases [5,6]. In a leaf, for example, at the early stages of development the cells within leaf primordia undergo a period of cell proliferation and differentiation; cell expansion subsequently begins at the leaf tip and proceeds progressively in a basipetal direction, a process during which the transition from cell proliferation to expansion is established [10-12]. Finally, the leaf grows by post-mitotic cell expansion and reaches its characteristic final size. Recent studies in Arabidopsis have suggested strongly that the timing of the transition from the cell proliferation to the cell expansion phase appears to be a critical determinant of overall organ size [12-15]. This notion is supported by the large number of identified factors involved in organ-size control that are known to alter the duration of cell proliferation, and thereby alter total cell number and final organ size [6-8]. For instance, some positive regulators that participate in organ-size control such as AINTEGUMENTA (ANT), AUXIN-REGULATED GENE INVOLVED IN ORGAN SIZE (ARGOS), and KLUH/CYP78A5 (KLU) can prolong the cell proliferation phase and thus increase cell numbers [9,16-18]. There are some negative regulators such as DA1 and ENHANCE OF DA1 (EOD1)/BIG BROTHER (BB), had a role in restricting the period of cell proliferation and thus the organ size [19-21]. Furthermore, factors involved in the regulation of cell expansion during organ growth have been identified in Arabidopsis, including the REGULATORY PARTICLE AAA-ATPASE 2a (RPT2a), EXPANSIN10 (EXP10), ARGOS-LIKE (ARL), and TARGET OF RAPAMYCIN (TOR) [22-26]. These factors were shown to affect either the duration or the rate of cell expansion, and are thus known to alter the final size of cells and of organs. Interestingly, there is a “compensatory mechanism” that coordinates cell proliferation and expansion during organ growth [27-29]. For instance, the *an3* mutant had fewer but larger cells than did wild-type plants, and further analysis indicated that the *an3* cells seemed to generate and transmit an intercellular signal that could enhance post-mitotic cell expansion [30]. It appears that when the cell number within an organ was decreased below a threshold level, the plants often triggered further post-mitotic cell expansion to compensate for the reduction in cell number [29,30]. The mechanisms responsible for the coordination of cell proliferation and expansion during plant organogenesis are far from being understood.

The Arabidopsis ORGAN SIZE RELATED (OSR) family has been implicated in the regulation of aerial organ size [17,23,31]. *ARGOS*, the founding member of the *OSR* homologues, controlled the growth of aerial organs, mainly through prolonging the duration of cell proliferation by promoting the continued expression of *ANT* and *CycD3;1* [17]. However, *ARGOS-LIKE* (*ARL*), a close homolog of *ARGOS*, was found to regulate the organ-size by affecting cell expansion [23]. Our recent work demonstrated that *ORGAN SIZE RELATED1* (*OSR1*) promoted organ growth by affecting both cell proliferation and expansion [31]. OSR1, ARGOS, and ARL all share a conserved OSR domain that is sufficient to promote organ growth [31]. Moreover, these three *OSR* genes are differentially regulated by various plant hormones, suggesting that they may mediate different signals to affect the cell proliferation and/or expansion process. Indeed, a recent study showed that *Zea mays ARGOS1* (*ZAR1*) had a function similar to that of *ARGOS*. Overexpression of *ZAR1* in maize enhanced maize organ growth, increased yield, and improved tolerance to drought stress [32]. *OSR* genes have only been identified in plants, and it is very interesting that different OSR members with a similar OSR domain could mediate cell proliferation and/or cell expansion, the two different cellular events that have been widely considered to be separately controlled [9,33,34].

There are four OSR homologues in the Arabidopsis genome, and these were likely generated by gene duplication events [31]. To gain further insight into the evolutionary function of the *OSR* genes, we investigated the role of the fourth *OSR* gene, *OSR2* (At2g41225) during organ growth. Here, we report that *OSR2* participates in the regulation of organ growth by primarily affecting cell expansion in a manner redundant with *ARL*. Further analysis of *osr2 argos-1 ARLi* triple and *argos-1 osr1 ARLi OSR2i* quadruple mutants revealed that these four *OSR* genes have redundant and cooperative roles in the regulation of cell proliferation and/or expansion. Our characterization of functional divergence among these four *OSR* genes implies a novel mechanism underlying the coordinative regulation of cell proliferation and expansion during plant organogenesis.

## Results

### *OSR2* is expressed primarily in organs undergoing growth by cell expansion

We previously reported that Arabidopsis *OSR1* was involved in the regulation of organ growth and that it contributes to final organ size primarily through enhancement of cell proliferation [31]. We also showed that three identified OSR proteins shared a conserved OSR domain with an identical LPPLPPPP motif and two putative transmembrane helices, which was sufficient to promote organ growth [31]. The *Arabidopsis thaliana* genome also has another gene, At2g41225, which encodes a protein of only 67 amino acids that phylogenetically belongs to the OSR member (Additional file 1: Figure S1A) [31]. Careful alignment of its amino acid sequence with those of OSR1, ARGOS, and ARL showed that At2g41225 had the conserved LPPLPPPP motif and the C terminal transmembrane helix of the OSR domain. However, it lacked the N terminal transmembrane helix but instead had a plasma membrane-localized signal peptide predicted by Phobius and iPSORT (Additional file 1: Figure S1B) (http://www.ebi.ac.uk//Tools/pfa/phobius; http://ipsort.hgc.jp). Moreover, At2g41225 is located alongside *OSR1* in the genome, suggesting that these two genes may originate from a gene duplication event [31]. Therefore, we designated At2g41225 as *Organ Size-Related 2* (*OSR2*).

**Figure 1:**
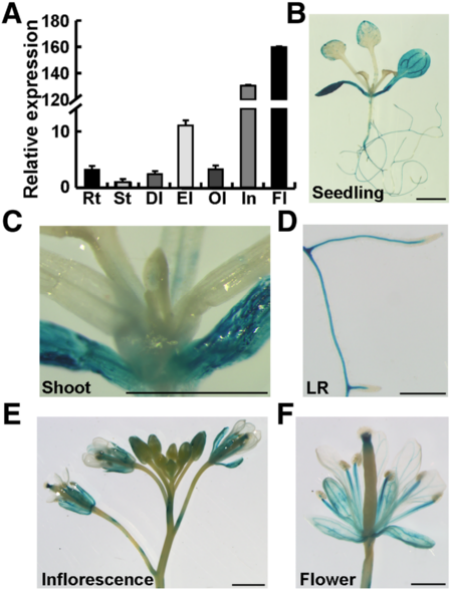
**Expression of** ***OSR2*** **. (A)** Expression of *OSR2* in various organs assayed by qRT-PCR. The data were from three biological replicates and are presented as mean values ± SE. Rt, root; St, stem; Dl, dividing leaf; El, expanding leaf; Ol, old leaf; In, inflorescence; Fl, flower. **(B-F)** Expression patterns of *OSR2* assayed by GUS staining of transgenic plants carrying a *proOSR2:GUS* construct. GUS staining was shown in seedling **(B)**, shoot **(C)**, lateral root (LR, **D**), and the floral organs **(E, F)**. Bars, 2 mm.

To explore whether or not *OSR2* is functional during organogenesis, we initially monitored *OSR2* expression in various organs with qRT-PCR analysis. As shown in Figure 1A, *OSR2* was expressed primarily in the expanding organs, including leaf, inflorescence, and flower. We then examined the tissue-specific expression of *OSR2* using the T3 independent transgenic plants carrying an *OSR2* promoter:β-glucuronidase (*proOSR2:GUS*) construct. In two-week-old seedlings, high GUS expression was observed in the organs or organ regions where active cell expansion and elongation were occurring, such as the upper parts of leaves, and the elongating regions of lateral roots (LR); abundant expression was also detected in the cotyledons (Figure 1B-D). However, weak or nearly absent GUS signals were observed in juvenile leaves, shoot, and root tips, where cells were predominantly undergoing the cell proliferation process (Figure 1B-D). In the inflorescence and developing floral organs, GUS staining was mainly detected in buds, sepals, and filaments (Figure 1E,F). Careful examination of GUS expression in developing first leaves showed that weak GUS signal was detected at the juvenile stage, while abundant GUS staining was observed when a leaf was undergoing expanding growth. Subsequently, the GUS signal was almost disappeared in fully-expanded leaves (Additional file 1: Figure S1C). These observations indicate that *OSR2* is expressed predominantly in organs that are undergoing cell expansion growth.

Since the three previously-identified OSR members were known to be ER-localized [31], we further examined the cellular localization of OSR2 in leaf epidermal cells of *proOSR2:OSR2-GFP* transgenic plants. Unlike other OSR proteins, the OSR2-GFP fusion protein signal was detected in the plasma membrane (Additional file 1: Figure S1D), indicating that OSR2 is plasma membrane-localized.

### Ectopic expression of *OSR2* enlarges organs primarily by enhancing cell expansion

**Figure 2:**
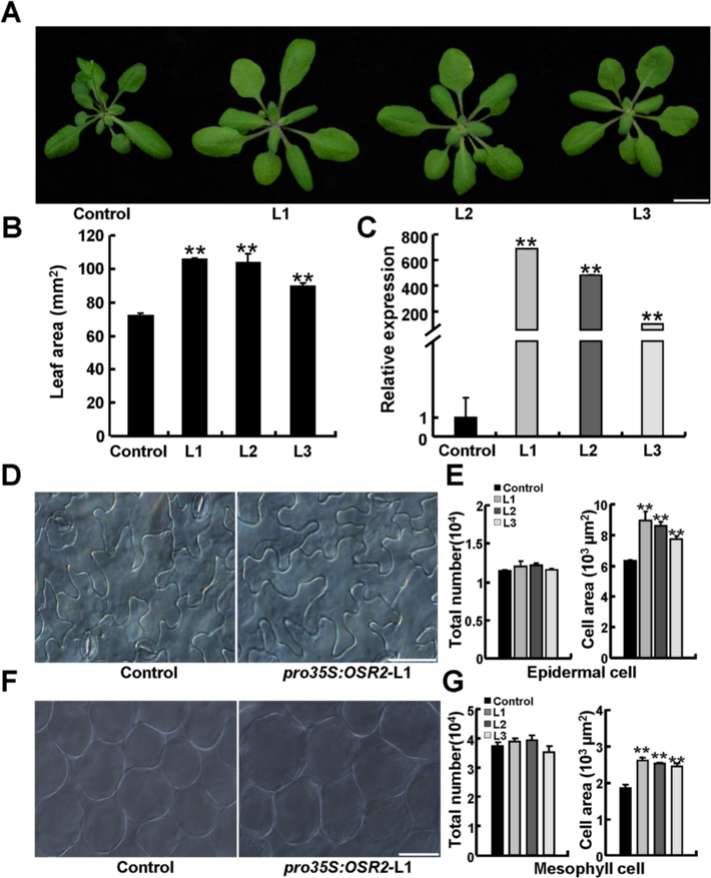
**Morphological and cytological characterization of** ***pro35S:OSR2*** **transgenic plants. (A)** Morphology of 28-day-old control and three independent lines of *pro35S:OSR2* transgenic plants (L1 to L3). Bar, 1 cm. **(B)** Blade areas of fully-expanded third leaves of control and three *pro35S:OSR2* transgenic lines. At least five leaves for each genotype were examined; data are shown as mean values ± SE; Student’s *t*-test: **P < 0.01. **(C)** qRT-PCR analysis of *OSR2* expression in control and transgenic plants presented in **(A)**. The data were from three biological replicates and are presented as mean values ± SE; Student’s *t*-test: **P < 0.01. **(D, E)** The cell area and estimated cell number of epidermal cells in fully-expanded third leaves of control and three *pro35S:OSR2* transgenic plants described in **(A)**. At least five leaves were examined for each line, and data are shown as mean values ± SE; Student’s *t*-test: **P < 0.01. Bar, 50 μm. **(F, G)** The cell area and estimated cell number of mesophyll cells in fully-expanded third leaves of control and *pro35S:OSR2* (three lines) transgenic plants. Bar, 50 μm. Data are shown as mean values ± SE; Student’s *t*-test: **P < 0.01.

To investigate the role of *OSR2* in organ development, we generated transgenic *Arabidopsis thaliana* (Col-0) plants harboring a *pro35S:OSR2* construct. All 35 of the T1 transgenic lines (independent transgenic events) overexpressing *OSR2* exhibited, to varying extents, enlarged organs as compared with the empty vector control plants. Careful examination of three of the independent T3 homozygous lines (L1, L2, L3) showed that the ectopic expression of *OSR2* resulted in an obviously increased size of various organs such as leaves, cotyledons, roots, floral organs, and siliques (Figure 2A, Additional file 1: Figure S2A, B). Detailed quantification of fully expanded third leaves revealed that the average blade areas of the *pro35S:OSR2* L1, L2, and L3 plants were increased by 46.8, 44.4, and 24.1%, respectively, as compared with those of control plants (Figure 2B). qRT-PCR analysis verified that the increased sizes of organs were indeed closely associated with the elevated *OSR2* expression in these transgenic lines (Figure 2C). The transgenic plants overexpressing *OSR2* also displayed longer roots, hypocotyls, siliques, and petioles, and the plant heights were also increased in these plants as compared with the controls (Additional file 1: Figure S2C-F). Additionally, the flowering time in *OSR2* transgenic plants was delayed by about four days (Additional file 1: Figure S2E).

We then used leaves and cotyledons as representative organs to investigate the contribution of cell proliferation and cell expansion to the enlarged organs of the *p35S:OSR2* plants. As shown in Figure 2D and E, as compared with those of the control leaves, the average size of the epidermal cells in fully-expanded third leaves of L1, L2, and L3 plants was increased by 42.3, 36.4, and 22.7%, respectively. The estimated numbers of epidermal cells per leaf were not obviously altered in the transgenic plants (Figure 2E). Similarly, mesophyll cells in *p35S:OSR2* leaves were enlarged, whereas the estimated cell numbers did not differ from the control leaves (Figure 2F,G). Consistently, ectopic expression of *OSR2* resulted in larger mesophyll cells in cotyledons, and again the mesophyll cell number in cotyledons did not differ from the control (Additional file 1: Figure S3A-C). These observations demonstrate that the enlarged organs in *p35S:OSR2* resulted primarily from enhanced cell expansion rather than cell proliferation.

### *OSR2* affects the cell expansion rate during organ growth

**Figure 3:**
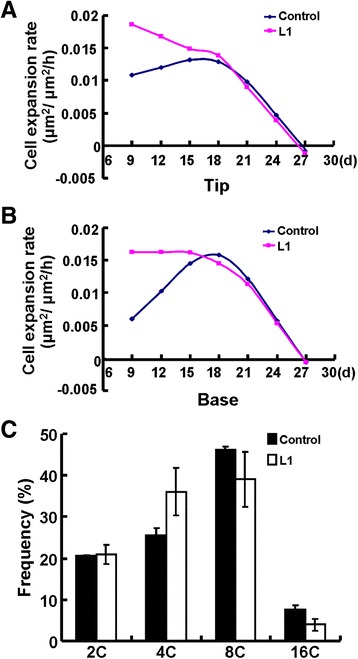
***OSR2*** **increases the rate of cell expansion in developing leaves. (A, B)** Relative cell expansion rate of the epidermal cells at the tip **(A)** and base **(B)** of the third leaves of control and *pro35S:OSR2* transgenic plants (L1). At least five leaves from each genotype were examined. **(C)** Nuclear polyploidization analysis of the leaf cells in the control and L1 *pro35S:OSR2* transgenic lines. Fully-expanded blades of the third leaves were used for cell nuclear ploidy analysis with a flow cytometer. The percentages of cells with different nuclear polyploidy levels were observed from three independent biological duplicates, and are shown as mean values ± SE.

To define the exact role of *OSR2* in cell expansion rate and duration, we further compared the expansion kinetics of epidermal cells in both the tips and the basal regions of the third leaves in control and *p35S:OSR2* L1 plants. As shown in Figure 3A and B, the cell expansion rates of both the tips and the bases of *p35S:OSR2* leaves were indeed much higher than those of controls from day 9 to day 16, whereas the cell expansion rates in the two genotypes were almost the same after day 21; cell expansion ceased after day 27(Figure 3A,B). These observations strongly suggest that ectopic expression of *OSR2* mainly affects the rate of cell expansion rather than the duration of cell expansion phase.

As cell endoreduplication is known to contribute to final cell size [35,36], we investigated whether the enlarged cells in the *p35S:OSR2* leaves were attributable to alteration of endoreduplication. Flow cytometry analysis of the nuclei of fully expanded leaves of 27-day-old plants showed that, although the percentages of cells from 4C and 8C were slightly different between the two genotypes, the overall distribution of cells from 2C to 16C remained comparable between two genotypes (Figure 3C). This result indicates that *OSR2*-mediated cell expansion is not related to the nuclear DNA endoreduplication of leaf cells.

### *OSR2* functions redundantly with *ARL* to mediate cell expansion

**Figure 4:**
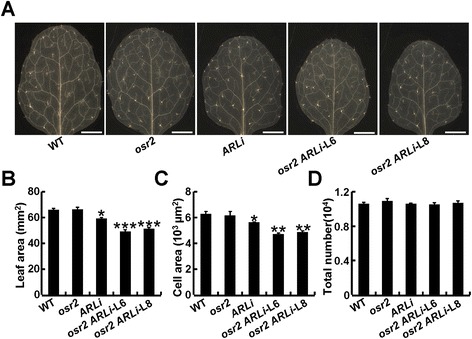
***OSR2*** **acts redundantly with** ***ARL*** **. (A, B)** Morphology and the size of third leave of 26-day-old WT, *osr2*, *ARLi,* and *osr2 ARLi* (L6 and L8) plants. At least five fully-expanded leaves of each genotype were used for determination of the leaf size; the data are shown as mean values ± SE; Student’s *t*-test: *P < 0.05, ***P < 0.001. Bars, 2 mm. **(C, D)** The epidermal cell area and estimated cell number of the third leave described in **(A)**. Data were from at least five leaves of each genotype and are shown as mean values ± SE; Student’s *t*-test: *P < 0.05, **P < 0.01.

To further investigate the role of *OSR2* in organ growth, we obtained the T-DNA insertion mutant *osr2* (Salk_142851, Col-0) from the Arabidopsis Biological Resource Center (ABRC), in which a T-DNA fragment was inserted into the exon of *OSR2* (Additional file 1: Figure S4A). RT-PCR analysis showed that *osr2* was an *OSR2* knock-out mutant (Additional file 1: Figure S4B). However, *osr2* plants didn’t show an obvious reduction in organ size when compared with wild-type (WT) plants (Figure 4A). Since the tissue-specific expression pattern of *OSR2* was quite similar to that of *ARL* (another OSR member that was reported to regulate organ growth by cell expansion [23]), we speculated that *OSR2* may have functional redundancy with *ARL*. To test this, we introduced an *ARL* specific *RNA-interference* construct (*pro35S: ARL RNAi*) into both WT and *osr2* plants to generate *ARLi* and *osr2 ARLi* plants. qRT-PCR analysis validated that the expression of *ARL* was obviously knocked down in the independent T3 lines (Additional file 1: Figure S4C, D). The final size of the third leaf of the *ARLi* transgenic plants was reduced by about 9% as compared with that of either the WT or *osr2*. By contrast, leaf size in *osr2 ARLi* plants L6 and L8 was decreased by about 24.4 and 22.7%, respectively (Figure 4B). Consistently, careful examination of the number and size of epidermal cells in the third leaf confirmed that the knockout of *OSR2* led to size reduction of epidermal cells in *ARLi* plants but had no obvious effect on cell number (Figure 4C,D). Consistently, transgenic plants harboring a 35S promoter-driven specific *OSR2 RNAi* construct (*pro35S:OSR2 RNAi*) did not exhibit any organ-size phenotype. However, introduction of a *pro35S:OSR2 RNAi* construct into *ARLi* plants enhanced the size reduction of leaves (Additional file 1: Figure S4E and F), confirming that *OSR2* acts redundantly with *ARL* in the regulation of cell expansion during organ growth.

As the three previously-identified *OSR* genes are known to be differentially responsive to various hormones [17,23,31], we also investigated the transcriptional regulation of *OSR2* by plant hormones. Interestingly, the qRT-PCR analysis of seedlings treated with various hormones revealed that, similar to *ARL*, *OSR2* was induced by epi-brassinolide (epi-BL) (Additional file 1: Figure S5A), and this induction was disrupted in the BR signaling mutants *bri1-6* and *bin2* (Additional file 1: Figure S5B). Moreover, introduction of a *p35S:OSR2* construct into *bri1-6* and *bin2* could partially restore leaf and petiole growth, and the expansion defect in *bri1-6* and *bin2* leaf cells was partially restored by overexpression of *OSR2* (Additional file 1: Figure S5C-F). These findings suggest that *OSR2* may also be involved in BR-related cell expansion, further supporting the likely functional redundancy between *OSR2* and *ARL* during organ growth.

### *OSR* genes cooperatively regulate cell proliferation and expansion during organogenesis

**Figure 5:**
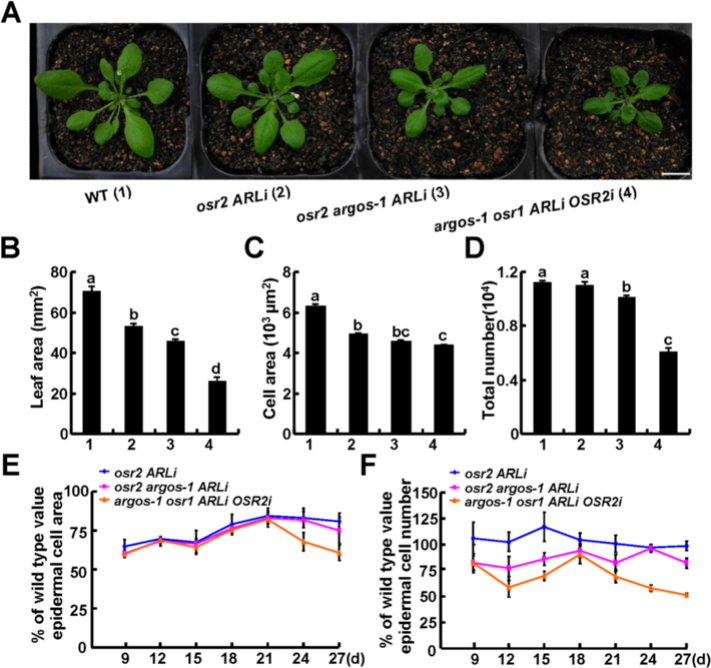
**Cooperative roles of** ***OSR2*** **and other** ***OSR*** **genes in cell proliferation and expansion. (A)** Morphology of 28-day-old WT, *osr2 ARLi, osr2 argos-1 ARLi,* and *argos-1 osr1 ARLi OSR2i* plants. Bar, 1 cm. **(B-D)** The leaf area **(B)**, epidermal cell area **(C)**, and estimated epidermal cell number **(D)** of the third leaves of the 30-day-old plants described in **(A)**. At least five leaves were examined for each genotype; the data are shown as mean values ± SE; the letters (a to d) indicate statistical significance (P < 0.05) among the genotypes according to one-way ANOVA testing (SPSS 13.0, Chicago, IL, USA). **(E, F)** The cellular kinetics of developing third leaves of WT and *osr* mutant plants. The epidermal cell size **(E)** and estimated epidermal cell number **(F)** are shown as the ratio of mutant/WT from 9–27 day-old plants. At least five leaves of each genotype were examined.

Our previous work revealed that *ARGOS* and *OSR1* regulated organ growth by primarily affecting cell proliferation in a redundant manner [31]. To examine the cooperative role of the OSR family members in organ growth, we generated an *osr2 argos-1 ARLi* triple mutant and an *argos-1 osr1 ARLi OSR2i* quadruple mutant by introducing a *pro35S:OSR2 RNAi* construct into *argos-1 osr1 ARLi* plants (Figure 5A). qRT-PCR analysis confirmed that the expression of *ARL* was knocked down in the *osr2 argos-1 ARLi* triple mutant, and the expression of both *ARL* and *OSR2* transcripts was decreased in the *argos-1 osr1 ARLi OSR2i* quadruple mutant (Additional file 1: Figure S6A, B). As expected, the introduction of *argos* mutation to *osr2 ARLi* plants further reduced the final size of their organs, and this reduction was enhanced in the quadruple mutant *argos-1 osr1 ARLi OSR2i* plants (Figure 5A,B). Cytological analysis of epidermal cells of the third leaves in these plants indicated that this reduction was primarily caused by a decrease in cell numbers (Figure 5C,D), confirming the role of *ARGOS* and *OSR1* in cell proliferation. These differential roles of *OSR* genes in cell proliferation or expansion were further verified by examining the kinetics of cell proliferation and expansion during organ growth in these genotypes (Figure 5E,F). Notably, we observed that epidermal cells of leaves in the *OSR* quadruple mutant were slightly smaller than those of the *osr2 ARLi* plants (Figure 5C,E). This is consistent with the previous finding that *OSR1* had some effects on cell expansion during organogenesis [31].

## Discussion

### Characterization of *OSR2* defines a new OSR member involved in the regulation of organ growth

The development of an organ relies on the coordination of cell proliferation and expansion; these two different cellular events determine the overall organ size. Cell proliferation, which sets the cell number of an organ, plays an important role in organ growth and final size control [8]. Post-mitotic cell expansion, which determines the final cell size, is also critical for organ development and size regulation [37]. Recently, several genes that participate in the regulation of cell expansion and thus cell size have been also reported to affect final organ size, such as *EXP10*, *RPT2a*, *TOR*, *ARL*, and *ErbB-3 EPIDERMAL GROWTH FACTOR RECEPTOR BINDING PROTEIN 1* (*EBP1*) [8]. Here, we showed that *OSR2* participates in the regulation of cell expansion during organ growth. *OSR2* was expressed in the organs or tissues undergoing cell expansion and elongation, and ectopic expression of *OSR2* increased overall organ size by enhancing the cell expansion rate. Although the loss-of-function mutant of *OSR2* had no obvious phenotype, our detailed examination revealed that *OSR2* acted redundantly with *ARL*, another OSR member that had been shown previously to affect cell expansion and thus final organ size [23]. Consistently, the expression pattern of *OSR2* in various organs and the response of *OSR2* expression to hormone treatment were quite similar to those of *ARL*, supporting the supposition that these genes have functional redundancy in organ growth. Therefore, our work identified *OSR2* as a new regulator of plant organ growth and final organ size, thereby providing an alternative to use *OSR2* in efforts to manipulate biomass production in plants.

### OSR-mediated organ growth may represent an evolutionary mechanism of the cooperative regulation of cell proliferation and expansion

There are four *OSR* genes in the Arabidopsis genome, and phylogenetic analysis showed that these four genes likely originated from gene duplication events. *ARGOS* and *ARL* were clustered into a group and *OSR1* and *OSR2* were clustered into a separate group [31]. *OSR1* and *OSR2* are located adjacent in the Arabidopsis genome. Surprisingly, *ARGOS* and *OSR1* are expressed predominately in organs or tissues undergoing growth by cell proliferation, and they are known to act upstream of *ANT* and redundantly regulate the duration of cell proliferation and thus the final organ size [17,31]. *OSR2* and *ARL* are expressed primarily in organs and tissues in which cell expansion/elongation is occurring, and our work here demonstrates that these genes mediate the cell expansion process and thus organ size. It is interesting that the different members of a co-evolved family function differentially in the regulation of cell proliferation or expansion, two fundamental cellular events that have long been considered to be separately controlled in multicellular organisms [9,33,34]. Therefore, OSR-mediated organ growth might represent an evolved mechanism of cooperative control of cell division and expansion at the organ level. Since OSRs were identified only in plants, and *OSR* mutations only resulted in organ growth phenotypes, it is likely that the OSR regulatory pathway represents a mechanism with which plants respond to growth signals or environmental cues to modify their growth and final organ size. Indeed, the *OSR* genes were transcriptionally regulated by treatment with different hormones. *ARGOS* expression is known to be induced by auxin and cytokinin; *OSR1* expression is induced by ethylene, but repressed by ABA and BR; the expression of *ARL* and *OSR2* is induced by BR [17,23,31]; *Zea mays ARGOS1* (*ZAR1*) is known to regulate the final size of maize organs and thus contributes to increased yields, particularly in drought stress conditions [32].

### The molecular roles of OSR family members during plant organogenesis

Our characterization of OSR members raises a critical question: how do the different members of the OSR family participate in the regulation of two different cellular events? More importantly, although cell division and cell expansion are strictly coordinated at both the cellular and organ levels, the two processes are generally considered to be controlled by the different mechanisms [9,33,34]. There has been little evidence so far that these two events share the same molecular machinery. Moreover, our previous work demonstrated that the OSR domain was sufficient to promote the organ growth, and that OSR1 also had some effect on the cell expansion process [31]. Obviously, the involvement of different *OSR* genes in cell proliferation and/or expansion relies on their temporal-spatial expression in developing organs; *ARGOS* and *OSR1* are expressed primarily in the cell proliferation phase while *ARL* and *OSR2* are expressed primarily in the cell expansion phase [17,23,31]. It still remains unclear how they facilitate the regulatory role in cell division or expansion with a similar OSR domain. Our previous work showed that three OSR proteins were likely localized to the ER, and here we showed that OSR2 is localized to plasma membrane. We still do not know whether the ER-localized OSR proteins or the plasma membrane-localized OSR2 are functional or processing forms. Because OSRs are small proteins, we also cannot exclude the possibility that OSRs may be functional as peptide signals. Finally, it is more likely that different OSR members may differentially interact with the variety of proteins, which would in turn diverge the roles of OSR in cell proliferation or expansion. Therefore, it will be critical to identify proteins that interact with OSR proteins and to dissect and characterize their downstream targets. Such efforts will be necessary for elucidating the molecular mechanism underlying OSR-mediated cell proliferation and expansion at the organ level.

## Conclusions

In conclusion, we revealed that the Arabidopsis *OSR2* participates in regulation of cell expansion during organogenesis and thus the organ size. Our findings of the differential and cooperative roles of *OSR* genes in regulation of cell proliferation or expansion strongly suggest that OSR-mediated organ growth may represent an evolutionary mechanism of the cooperative regulation of cell proliferation and expansion during plant organogenesis.

## Methods

### Plant materials and growth conditions

*Arabidopsis thaliana* ecotype Columbia (Col-0) was used in this study. *osr2* (Salk_142851, Col-0) and *argos-1* (SAIL_896_G10, Col-0) were obtained from the Arabidopsis Biological Resource Center (ABRC), and *osr1* (GABI_436G04) was from the Nottingham Arabidopsis Stock Centre (NASC) (http://www.arabidopsis.org). All seeds were sterilized in 0.5% sodium hypochlorite for 15 minutes, and geminated on 1/2 Murashige and Skoog (MS) medium in a culture room at 22°C under a 16-h light/8-h dark photoperiod with an illumination intensity of 80–90 μmol m^−2^ s^−1^. Seven-day-old seedlings were transferred to soil and grown in a growth room at 22 ± 1°C, under the same photoperiod and illumination regime as those in the culture room [38].

### Sequence alignment

The full length amino acid sequences of OSR2, OSR1, ARGOS, and ARL were obtained from The Arabidopsis Information Resource (TAIR) database. Alignment analysis of the four proteins was performed with the MUSCLE program (http://www.ebi.ac.uk/Tools/msa/muscle/), and manually optimized with Genedoc software [39,40]. The cluster of four proteins was analyzed with CLUSTALW2 (http://www.ebi.ac.uk/Tools/msa/clustalw2).

### Plasmid construction and Arabidopsis transformation

To generate the *pro35S:OSR2* and *proOSR2:OSR2-GFP* constructs, the coding sequence of *OSR2* was amplified by RT-PCR and ligated into the pEASY-Blunt vector (TransGen Biotech, China) and sequenced. The *OSR2* fragment digested with appropriate restriction endonuclease(s) was cloned into the pVIP96 or pMDC83 plasmid [17,41]. For the *proOSR2:GUS* and *proOSR2:OSR2-GFP* construct, a 1880-bp genomic fragment from the *OSR2* promoter was cloned into the pMDC163 vector and the pMDC83 plasmid containing the coding sequence of *OSR2* [41]. For the RNAi constructs, a 142-bp cDNA fragment specific for *ARL* or a 193-bp cDNA fragment specific for *OSR2* was cloned into the pBluescript SK plasmid containing an RNAi fragment in both the sense and the antisense orientations [42], and then cloned into pVIP96 or pMDC83 to generate *pro35S:ARL RNAi* or *pro35S:OSR2 RNAi* constructs, respectively. All primers used in the generation of these constructs are detailed in Additional file 1: Table S1.

All generated constructs were introduced into *Arabidopsis thaliana* ecotype Columbia (Col-0) by *Agrobacterium tumefaciens*–mediated transformation via the described floral dip method [43]. For each construct, a minimum of 18 independent lines harboring a single T-DNA insertion were generated, and three independent lines of their T3 generation plants were used for detailed analysis.

### Hormone treatment and gene expression analysis

For the hormone treatment, nine-day-old seedlings were transferred to 1/2MS liquid medium containing 5 μm IAA, 5 μm kinetin (KT), 100 μm GA3, 5 μm ACC, 50 μm ABA or 1 μm 24-epi-brassinolide (epi-BL) for 3 h. Total RNA from different materials was isolated using TRIzol reagent (Invitrogen). After digestion with DNaseI, RNA was reverse transcribed with Superscript-III reverse transcriptase (Invitrogen) into cDNA for subsequent RT-PCR or qRT-PCR analysis. The transcript abundance of *GLYCERALDEHYDE-3-PHOSPHATE DEHYDROGENASE C SUBUNIT* (*GAPC*) or *ACTIN2* was used as an internal control in the RT-PCR or qRT-PCR analysis, respectively. The qRT-PCR analysis was performed with SYBR Premix Ex Taq Mix on a Rotor-Gene3000 instrument (Corbett Research) with three biological replicates, according to the manufacturer’s instructions. The primers used for expression analyses are detailed in Additional file 1: Table S1.

Seedlings or individual organs of homozygous transgenic plants carrying a *proOSR2:GUS* construct were used for the GUS staining assay. These were incubated in a 50 mM sodium phosphate solution (pH7.0) containing 5 mM K4Fe(CN)6, 5 mM K3Fe(CN)6, 0.1% Triton X-100, and 1 mM 5-bromo-4-chloro-3-indolyl-b-glucuronic acid (Gluc) at 37°C for several hours [31].

### Morphological and cytological analyses

For morphological characterization, six-day-old etiolated seelings grown in the dark were used for the measurement of the hypocotyl length; eight-day-old seedlings were used for the measurement of cotyledon area and root length; 28-day-old plants were used for the measurement of petiole length; 50-day-old plants were used to determine plant height and silique length.

To determine cell size and number, expanding or fully-expanded third leaves and cotyledon were excised and placed in a destaining solution for 30 minutes or overnight at room temperature. Then the destaining solution was exchanged with basic solution for 15–20 minutes at room temperature. The materials were rehydrated via an ethanol series for 10–15 minutes and were then transferred in water to visualize and measure cells [44]. The mesophyll and epidermal cells at the tip, central, and basal positions of a leaf were visualized under a microscope and photographed (Olympus BX51), and the areas of leaves and cells were measured with ImageJ 1.4.3.67 software (http://rsb.info.nih.gov/ij/). The total cell number per leaf was estimated as the total leaf area multiplied by the average cell number per area. To determine the cell expansion rate, the measured size of epidermal cells in leaves at different stages were transferred into log_2_ values and then fitted with a local five point quadratic function, the first derivative of which was calculated according to the method described by Nelissen *et al*. [45].

### Flow cytometric assay and confocal microscopy

Fully-expanded third leaves of control and transgenic plants overexpressing *OSR2* at 27 Day After Germination (DAG) were excised with a razor and then suspended in cold nuclear isolation buffer [46]. Flow cytometric analysis was carried out as described previously with a FACS Caliber flow cytometer (BD Biosciences, http://www.bdbiosciences.com/).

To examine the cellular localization of OSR2, the epidermal cells of transgenic plants harboring a *proOSR2:OSR2-GFP* construct were used to visualize GFP signals under a confocal microscope (Leica). FM4-64 staining was used to confirm the plasma membrane localization of the OSR2-GFP fusion protein.

### Accession numbers

The sequence data for the genes mentioned in this work can be found in Arabidopsis Information Resource (TAIR) database as: *OSR2* (At2g41225), *OSR1* (At2g41230), *ARL* (At2g44080), *ARGOS* (At3g59900), *GAPC* (At3g04120) and *ACTIN2* (At3g18780).

### Availability of supporting data

The data supporting the results of this article are included within the article and its additional file.

## Additional file

Additional file 1: Figure S1. Cluster and alignment of OSR2 and OSR proteins. **Figure S2.** Phenotypes of *p35S:OSR2* transgenic plants. **Figure S3.** Cytological characterization of cotyledons in *p35S:OSR2* transgenic plants. **Figure S4.** Characterization of *osr2* and *osr* mutants. **Figure S5.** *OSR2* is involved in BR-mediated cell expansion. **Figure S6.** Molecular characterization of *osr* mutants. **Table S1.** The primers used in this study.

## Abbreviations

ABRC: The Arabidopsis biological resource center
DAG: Day after germination
epi-BL: Epi-brassinolide
GUS: β-glucuronidase
KT: Kinetin
LR: Lateral roots
MS: Murashige and Skoog
OSR: ORGAN SIZE RELATED
RNAi: RNA-interference
TAIR: The Arabidopsis information resource
TOR: The target of the rapamycin
WT: Wild-type
