# Supplementary material for: The Arabidopsis ORGAN SIZE RELATED 2 is involved in regulation of cell expansion during organ growth
Source: BMC Plant Biol. 2014 Dec 10;14:349. doi: 10.1186/s12870-014-0349-5 (PMC4271509; doi:10.1186/s12870-014-0349-5)
Supplement: Additional file 1: Figure S1. — Cluster and alignment of OSR2 and OSR proteins. Figure S2. Phenotypes of p35S:OSR2 transgenic plants. Figure S3. Cytological characterization of cotyledons in p35S:OSR2 transgenic plants. Figure S4. Characterization of osr2 and osr mutants. Figure S5. OSR2 is involved in BR-mediated cell expansion. Figure S6. Molecular characterization of osr mutants. Table S1. The primers used in this study. [file 12870_2014_349_MOESM1_ESM.pdf]

Additional file

Qin Z, Zhang X, Zhang X, Feng G, Hu Y.

# The Arabidopsis *ORGAN SIZE RELATED 2* is involved in regulation of cell expansion during organ growth

**Figure S1**

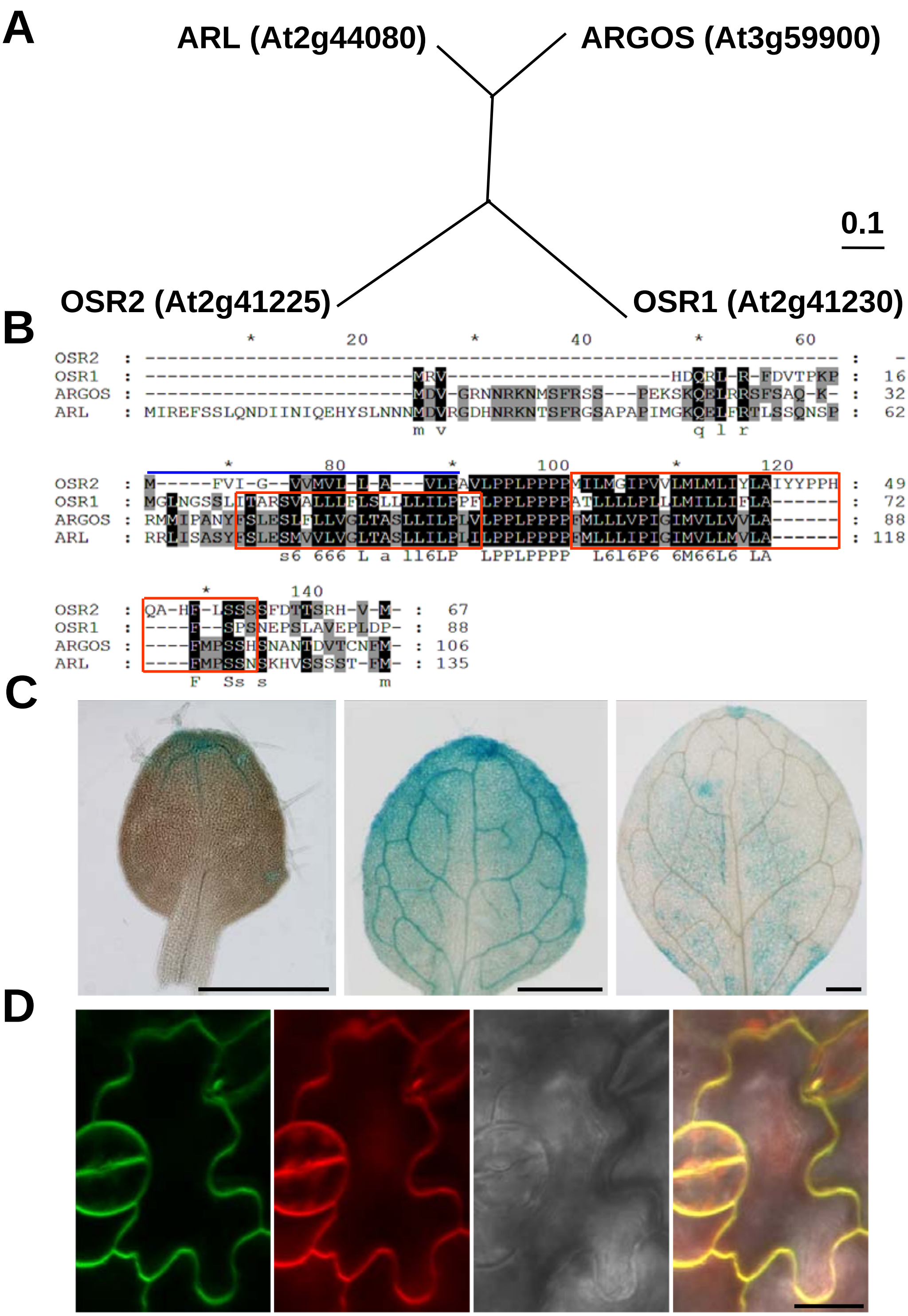


**Additional file 1: Figure S1** Cluster and alignment of OSR2 and OSR proteins.

(A) Cluster of OSR2, OSR1, ARGOS, and ARL. (B) Alignment of OSR2 and OSR proteins. Shading types represent different levels of amino acid identity. The blue line represents the plasma membrane-localized signal peptide of OSR2 predicted by Phobius. Red boxes indicate the conserved OSR domain. (C) GUS staining of the first leaves of transgenic plants carrying a *proOSR2:GUS* construct. The leaves of six-day-old, 16-day-old, and 20-day-old seedlings (from left to right) were assayed. Bars, 0.5 mm. (D) Subcellular localization of OSR2. Epidermal cells of transgenic plants carrying a *proOSR2:OSR2-GFP* construct are used to determine fusion protein localization. The GFP signal, FM4-64 signal (indicating the plasma membrane-localized), bright-field and overlaid images are shown from left to right. Bar, 10 μm.

**Figure S2**

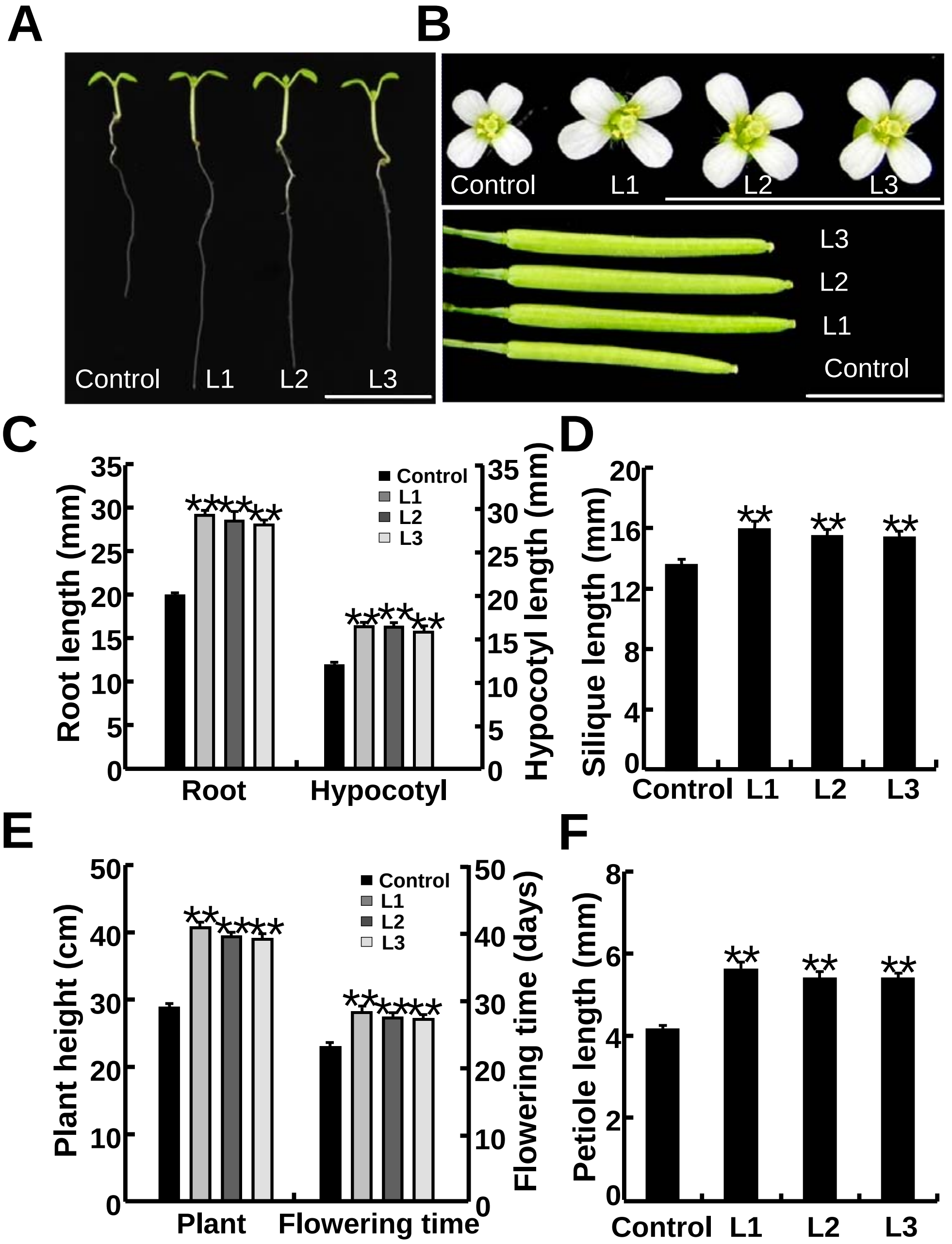


**Additional file 1: Figure S2** Phenotypes of *p35S:OSR2* transgenic plants.

(A, B) Morphology of eight-day-old seedlings (A), floral organs and siliques (B) of control and three independent *p35S:OSR2* transgenic lines (L1 to L3). Bars, 1 cm. (C-F) Root and hypocotyl length (C), silique length (D), plant height and flowering time (E), and petiole length (F) in control and the *p35S:OSR2* transgenic lines. Data are shown as mean values ± SE; Student's *t*-test: **P<0.01.

**Figure S3**

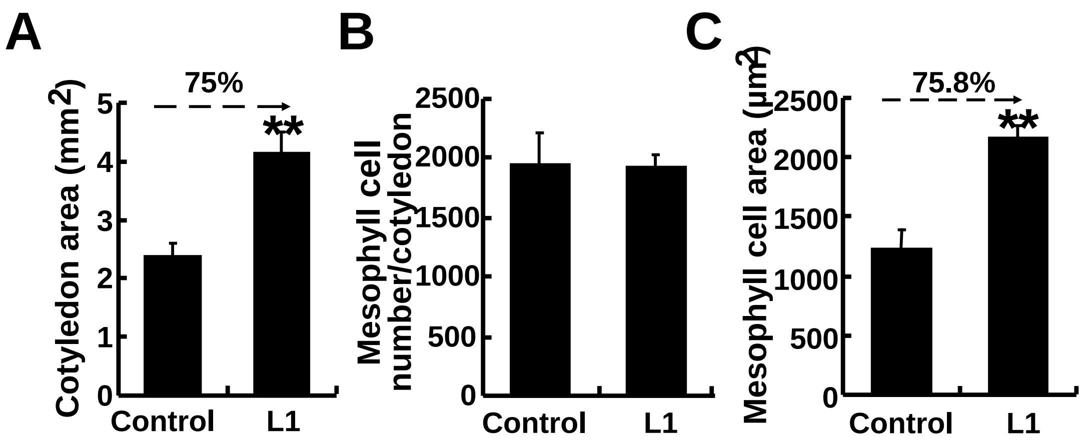


**Additional file 1: Figure S3** Cytological characterization of cotyledons in *p35S:OSR2* transgenic plants.

(A) Cotyledon area of eight-day-old seedlings in control and *p35S:OSR2* transgenic line, L1. (B, C) The estimated cell number (B) and cell area (C) of mesophyll cells in control and *p35S:OSR2* transgenic cotyledons. At least six cotyledons for each genotype were examined, and data are shown as mean values ± SE; Student's *t*-test: **P<0.01.

Figure S4

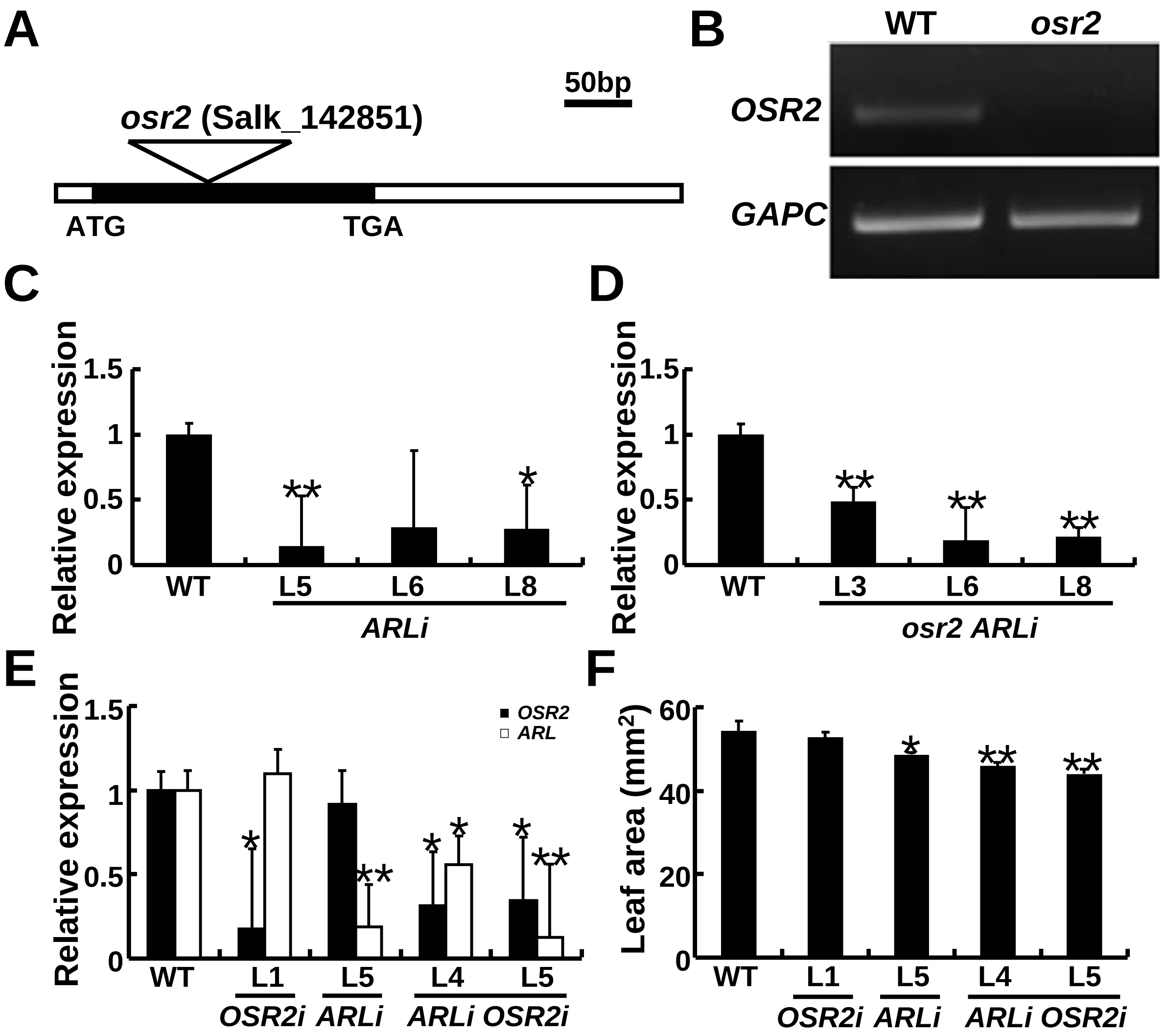


**Additional file 1: Figure S4** Characterization of *osr2* and *osr* mutants.

(A) Schematic illustration of the genomic region at the *OSR2* locus in *osr2* (Salk_142851). The coding region of *OSR2* is indicated as black box, and the UTR as white box. (B) *OSR2* expression in WT and *osr2* mutant. (C, D) qRT-PCR analysis of *ARL* expression in the independent *ARLi* lines (C) and *osr2 ARLi* lines (D). The data were from three biological replicates and are shown as mean values ± SE; Student's *t*-test: *P<0.05, **P<0.01. (E) qRT-PCR analysis of *OSR2* and *ARL* expression in WT, *OSR2i* line 1 (L1), *ARLi* line 5 (L5) and *ARLi OSR2i* plants (L4, L5). Data are shown as mean values ± SE; Student's *t*-test: *P<0.05, **P<0.01. (F) Blade areas of the third leaves of 25-day-old plants described in (E). At least five leaves were examined for each genotype; the data are shown as mean values ± SE; Student's *t*-test: *P<0.05, **P<0.01.

Figure S5

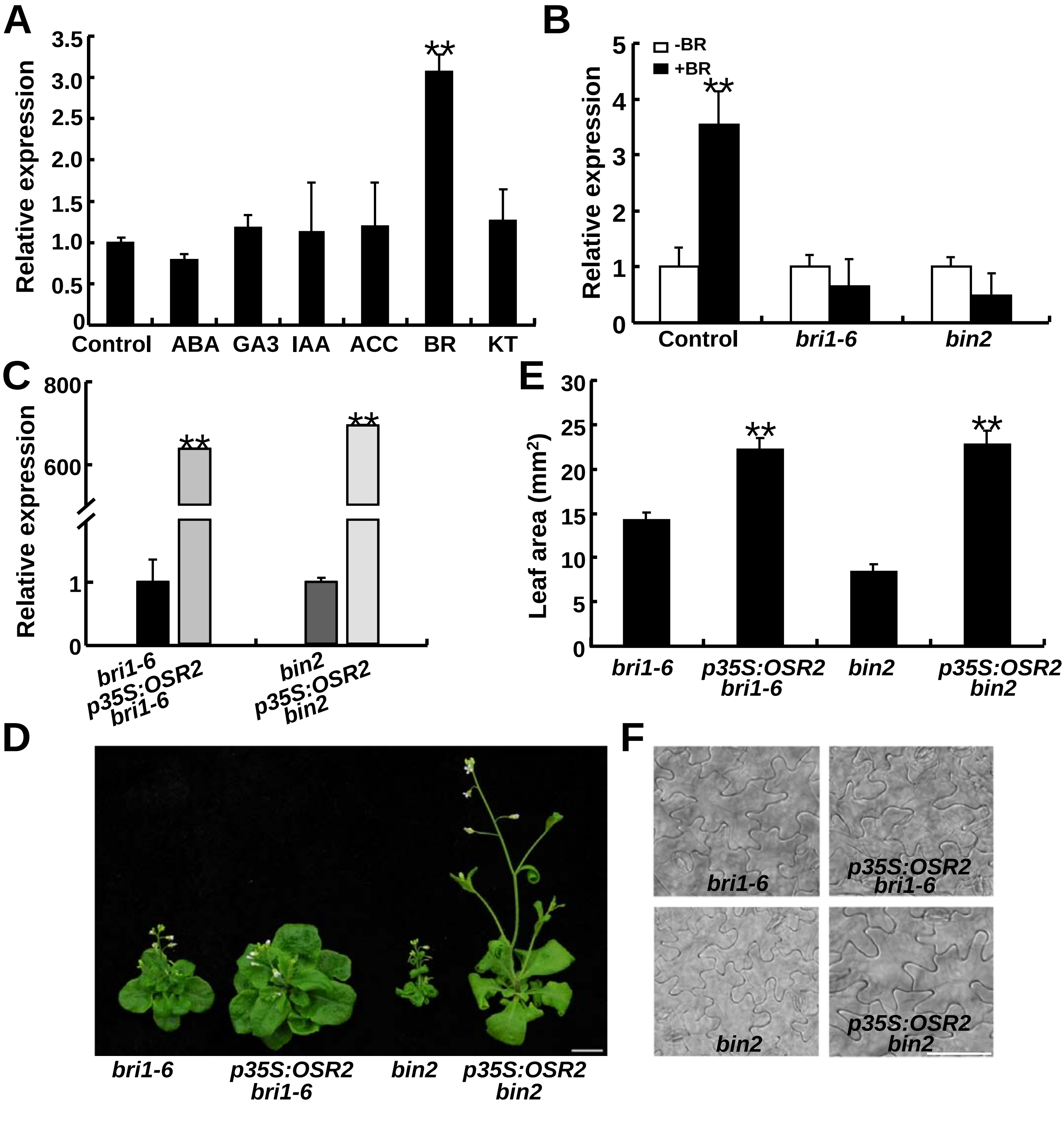


**Additional file 1: Figure S5** *OSR2* is involved in BR-mediated cell expansion.

(A) The transcriptional regulation of *OSR2* by plant hormones. The data were from three biological replicates and are presented as mean values ± SE; Student's *t*-test: **P<0.01. (B) *OSR2* induction by BR is disrupted in BR mutant *bri1-6* and *bin2.* (C) Expression of *OSR2* in *bri1-6*, *bin2*, *pro35S:OSR2 bri1-6*, and *pro35S:OSR2 bin2* plants*.* (D, E) The morphology (D) and leaf area (E) of 30- day-old plants described in (C). At least five leave for each genotype were examined, and the data are shown as mean values ± SE; Student's *t*-test: **P<0.01. Bar, 1 cm. (F) Epidermal cells of the four genotypes described in (C), Bar, 50μm.

**Figure S6**

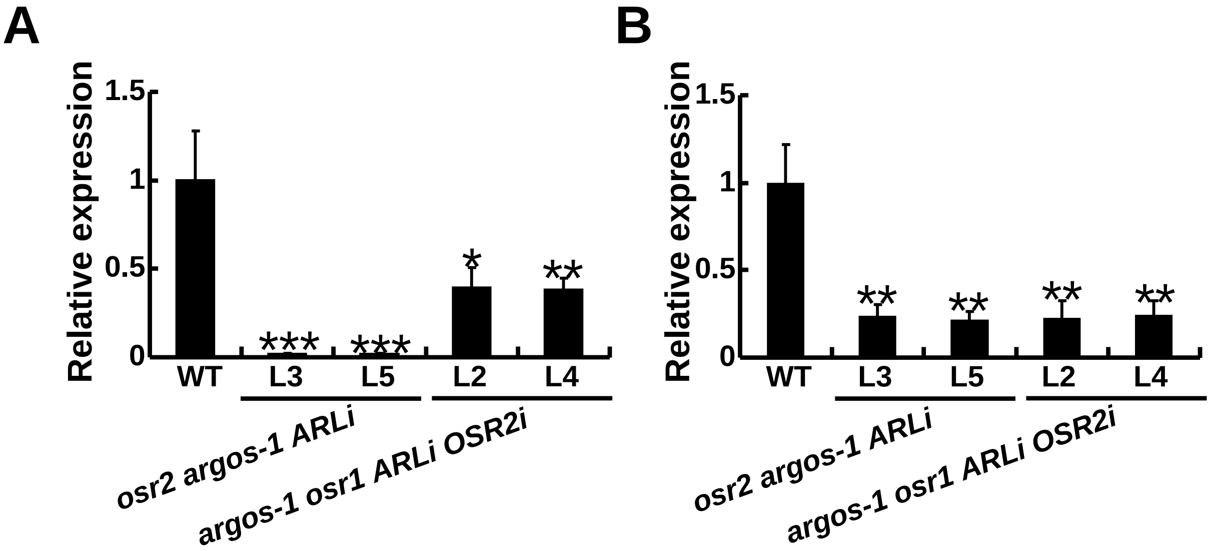


**Additional file 1: Figure S6** Molecular characterization of *osr* mutants.

(A, B) qRT-PCR analysis of *OSR2* (A) and *ARL* (B) expression in WT, *osr2 argos-1 ARLi* (L3, L5), and *argos-1 osr1 ARLi OSR2i* plants (L2, L4). Data are from three biological replicates and are shown as mean values ± SE; Student's *t*-test: *P<0.05, **P<0.01, ***P<0.001.

## Additional file1: Table S1. The primers used in this study.

| Primers | Sequences (5'-3') | Description |
|---|---|---|
| OSR2F | tctagaATGTTTGTGATTGGAGTGGTGA | *pro35S:OSR2* construct and RT-PCR |
| OSR2R | gaattcTCACATTACATGCCTAGAAGTAGTGT | |
| OSR2-GFP-F | actagtATGTTTGTGATTGGAGTGGTGA | *proOSR2:OSR2-GFP*construct |
| OSR2-GFP-R | ggcgcgccaCATTACATGCCTAGAAGTAG | |
| OSR2-GUS-F | ttaattaaTTTGTCTTCGCTTTATCGGGTCT | *proOSR2:GUS* construct |
| OSR2-GUS-R | ggcgcgccTTCGACGATCCTTTCGTATGTAAAT | |
| OSR2-FF | tctagaTTGACACTACTTCTAGGCATGTAATGTG | *pro35S:OSR2RNAi* construct-RNAi fragment |
| OSR2-FR | ctgcagTCCAACTCGAATCACCCTAAAACG | |
| OSR2-RF | ctcgagTTGACACTACTTCTAGGCATGTAATGTG | |
| OSR2-RR | atcgatTCCAACTCGAATCACCCTAAAACG | |
| ARL-FF | tctagaCTGATTTTCCAGATCAAACTTCGA | *pro35S:ARLRNAi* construct-RNAi fragment |
| ARL-FR | ctgcagGTTATGATCTCCTCTCACGTCCATG | |
| ARL-RF | ctcgagatctCTGATTTTCCAGATCAAACTTC | |
| ARL-RR | atcgatGTTATGATCTCCTCTCACGTCCATG | |
| OSR2-QF | TGATGGTGCTATTGGCGGTTCT | qRT-PCR |
| OSR2-QR | AGCATTAGCATCAGCACCACCG | |
| ARL-QF | TTCATCGTCAGGGTTCTT | qRT-PCR |
| ARL-QR | TCGAAGTTTGATCTGGAA | |
